# Artificial Intelligence-Enabled Ingredient Substitution in Food Systems: A Review and Conceptual Framework for Sensory, Functional, Nutritional, and Cultural Optimization

**DOI:** 10.3390/foods14223919

**Published:** 2025-11-17

**Authors:** Emel Oz, Fatih Oz

**Affiliations:** 1 Department of Food Engineering, Faculty of Agriculture, Ataturk University, Erzurum 25240, Türkiye; emel.oz@atauni.edu.tr; 2 East Anatolian High Technology Research and Application Center (DAYTAM), Ataturk University, Erzurum 25240, Türkiye; 3 Department of Food Engineering, Engineering Faculty, Kyrgyz-Turkish Manas University, Bishkek 720038, Kyrgyzstan

**Keywords:** ingredient substitution, artificial intelligence, flavor prediction, functional property modeling, nutritional profiling, constraint-based formulation, explainable AI, multi-objective food design

## Abstract

Ingredient substitution has become a multidimensional challenge in modern food systems, where sensory authenticity, functional performance, nutritional equivalence, and cultural or regulatory compliance must be satisfied simultaneously. This review examines how artificial intelligence (AI) can contribute to this problem by synthesizing current advances across four scientific domains relevant to substitution: flavor perception, matrix functionality, nutrient bioavailability, and socio-regulatory constraints. The review follows a narrative, domain-focused approach rather than a systematic or quantitative protocol, with literature selected from Scopus, Web of Science, and Google Scholar to capture both foundational food science studies and emerging AI applications. A modular framework for AI-enabled ingredient substitution is proposed and structured around four domains: (1) flavor and aroma modeling, (2) functional property prediction, (3) nutritional profiling, and (4) constraint-based filtering. The framework brings together a range of AI techniques—including machine learning, graph neural networks, natural language processing, and multi-objective optimization—and connects them to domain-specific datasets such as volatile compound libraries, rheological measurements, dietary reference databases, and regulatory ontologies. The review identifies three major gaps limiting real-world deployment: the lack of multimodal datasets linking composition, perception, and processing; limited explainability of current AI models; and weak integration between computational outputs and regulatory or industrial workflows. Addressing these barriers will be essential for developing transparent, scalable, and context-aware substitution systems that align with future directions in sustainable and personalized food innovation.

## 1. Introduction

Ingredient substitution refers to replacing one or more components in a formulation while preserving sensory quality, technological functionality, nutritional value, and cultural or dietary compatibility [1]. This need arises in diverse contexts, including allergen management, dietary preferences shifts, supply chain disruptions, and product reformulation. Unlike conventional ingredient replacement, substitution rarely has a one-to-one analogue and often requires compensatory adjustments to restore flavor, aroma, texture, emulsification, stability, and processing behavior.

Global transformations in food systems—including the rise of plant-based diets, the prevalence of food allergies, and growing demand for clean-label and ethically aligned products—have intensified the need for systematic substitution strategies [2]. Although plant-based formulations can reduce environmental impact, they also introduce challenges such as incomplete amino-acid profiles, altered sensory attributes, or micronutrient deficiencies [3,4]. In parallel, emerging ingredients increase the likelihood of novel allergenic responses [5], while personalized nutrition trends require substitutions that are not only functionally valid but also nutritionally targeted, culturally acceptable, and environmentally justified [6].

Despite these pressures, most substitution practices remain empirical, iterative, and difficult to scale. Trial-and-error methods struggle to optimize multiple competing objectives. This highlights the need for systematic, data-driven strategies grounded in a holistic understanding of ingredient behavior, sensory dynamics, and nutritional modeling [1,7]. However, the current literature on ingredient substitution remains fragmented across sensory, functional, and nutritional dimensions, lacking a unified theoretical framework. This fragmentation limits the scalability of innovation and underscores the need for an integrative synthesis.

Conventional substitution methods in food science are typically heuristic, relying on experience, formulation databases, or compositional similarity. While these can be effective for simple systems, they often fail to account for the complex interplay among sensory perception, matrix interactions, and nutritional impact [1]. These limitations become especially evident when substitutions must satisfy multiple objectives—such as replicating dairy fat mouthfeel, preserving emulsion stability, and meeting dietary restrictions [8]. Formulation toolkits and sensory panels offer some support, but they are qualitative, time-consuming, and poorly suited to iterative product development [7]. Moreover, the lack of integrated frameworks combining compositional, physicochemical, sensory, and nutritional data restricts the ability to model trade-offs and optimize substitution outcomes. As a result, substitution remains confined to narrow tasks—like sugar or fat replacement—rather than being treated as a dynamic, multidimensional design problem adaptable to shifting regulatory, economic, or consumer conditions. To overcome these limitations, researchers are turning to computational approaches—especially artificial intelligence (AI)—to support more robust, scalable decision-making.

The growing complexity of modern food formulation has accelerated interest in the use of artificial intelligence (AI) to address the multifaceted nature of ingredient substitution. AI systems—particularly those based on machine learning (ML), deep learning, and graph-based modeling—have demonstrated considerable promise in food science, especially for problems involving non-linear, high-dimensional, and interdependent variables. These technologies offer potential improvements in areas such as food production, quality control, safety, food security, nutrition supply chain management, and agrobusiness [9,10]. They can process heterogeneous datasets—including chemical compositions, sensory evaluations, recipe text corpora, and consumer preference data—to uncover latent patterns and generate predictive insights into ingredient behavior. Recent studies have shown that AI can support flavor pairing, recipe generation, nutritional optimization, and predictive modeling of texture and product stability [11,12,13,14]. Nevertheless, these advances remain largely dispersed, and no prior review has systematically connected AI methodologies to the multidimensional scientific basis of ingredient substitution.

However, the application of AI to fully integrated ingredient substitution—which requires balancing flavor compatibility, functional performance, nutritional equivalence, and socio-cultural constraints—remains fragmented and underdeveloped across disciplines [1,7,15]. Recent advances in explainable AI (XAI) address one of the most pressing limitations of food-related AI tools: the need for model transparency and interpretability [16,17]. These techniques enable the deconstruction of model decisions, thereby improving trust, regulatory compliance, and scientific scrutiny [18,19,20]. When combined with domain-specific food science knowledge, AI is increasingly positioned to move from a supportive analytical tool to a central framework for intelligent ingredient substitution.

Therefore, this review aims to synthesize the current state of AI applications in ingredient substitution, identify the major research gaps, and propose a multidimensional conceptual framework integrating sensory, functional, nutritional, and regulatory dimensions. By bridging data science and food science perspectives, the review provides a systems-level analysis that connects foundational principles, computational methodologies, modular architecture, and emerging applications in food innovation. Framing ingredient substitution as a multi-objective optimization problem, the review examines the emerging role of AI at the intersection of flavor chemistry, food functionality, nutritional design, and cultural or regulatory constraints. In contrast to previous literature, which often focused narrowly on flavor pairing or isolated reformulation strategies, this study offers a systems-level framework that captures the full complexity of modern food systems. The paper is structured across four key dimensions: (1) the scientific underpinnings of substitution, including volatile interactions, physicochemical properties, and dietary considerations; (2) state-of-the-art AI methodologies such as graph-based learning, recommender systems, and multimodal neural networks; (3) a proposed modular framework integrating flavor modeling, functional prediction, nutritional profiling, and constraint-based filtering; and (4) real-world applications in plant-based product design, allergen-free formulation, and circular ingredient valorization. Finally, the review highlights persistent gaps such as the lack of standardized sensory-labeled datasets, limited model generalizability, and weak integration of AI outputs into regulatory workflows, and it outlines a roadmap for developing transparent, scalable, and ethically responsible ingredient substitution systems.

## 2. Methodological Approach of the Review

This review aims to examine the emerging role of artificial intelligence (AI) in ingredient substitution within food systems. Rather than conducting a systematic or quantitative meta-analysis, the goal was to develop a conceptual and integrative synthesis that captures recent advances across sensory, functional, nutritional, and cultural dimensions of substitution. Relevant literature was identified through targeted searches in Scopus, Web of Science, and Google Scholar using topic-specific combinations of keywords related to AI, ingredient substitution, and food formulation. This flexible yet structured search strategy ensured the inclusion of scientifically grounded and interdisciplinary sources, while also allowing room to incorporate recent and rapidly developing contributions in this fast-evolving research area.

## 3. Scientific Foundations of Ingredient Substitution

**Figure 1:**
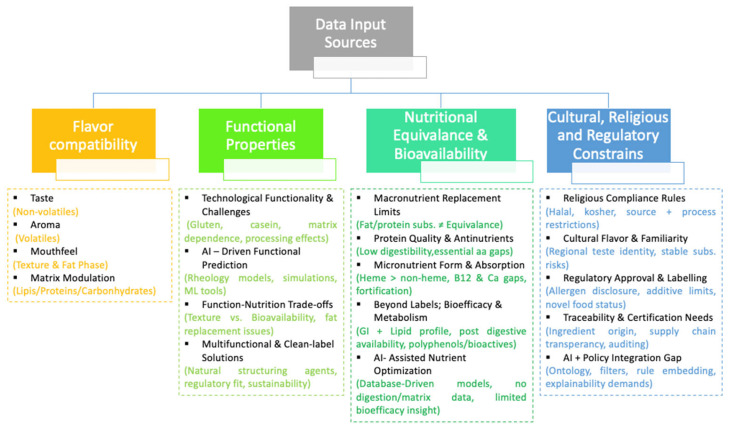
Multidimensional framework of factors influencing ingredient substitution feasibility.

Ingredient substitution is not a single-parameter problem but a multidimensional design challenge shaped by flavor compatibility, functional properties in food, nutritional equivalence and bioavailability, and cultural, religious, and regulatory constraints. Figure 1 provides an integrative overview of the core domains that collectively determine substitution feasibility.

### 3.1. Flavor Compatibility

Flavor compatibility is central to ingredient substitution because flavor perception is one of the strongest drivers of consumer acceptance. Flavor arises from the interaction of taste (non-volatile compounds), aroma (volatile compounds), and mouthfeel, all of which are influenced by the food matrix [21]. Even minor changes in volatile or non-volatile profiles can noticeably alter sensory quality and reduce acceptance. Aroma replication is especially difficult because odorants interact non-linearly, and trace-level molecules may exert a dominant sensory effect [22]. Matrix components such as lipids, proteins, and carbohydrates further modulate aroma release and perception [23]. For this reason, successful substitution requires more than chemical similarity—it requires understanding how a compound behaves within the physicochemical and structural context of the final product. Chemoinformatics tools and molecular similarity models are increasingly used to predict flavor analogues [24], but they remain limited by incomplete aroma databases and low accuracy for cross-modal sensory interactions, such as sweetness-enhanced aroma or fat-driven mouthfeel [25]. Cultural perception adds another layer of complexity: a chemically suitable substitute may still fail if it conflicts with regional taste expectations or traditional food identity [26]. AI-based tools such as graph-based flavor networks and neural embedding models trained on recipe and sensory review datasets are now able to propose culturally and sensorially coherent substitutions [27], although empirical sensory validation is still required.

### 3.2. Functional Properties in Foods

Food ingredients play essential technological and structural roles such as emulsification, foaming, gelation, water binding, and texture formation—key determinants of product stability and consumer acceptance [28]. Substitution becomes especially challenging when an ingredient provides matrix integrity; any replacement must perform comparably under variable processing conditions [28].

#### 3.2.1. Technological Functionalities and Challenges

In bakery systems, gluten ensures gas retention and viscoelasticity. Replacing gluten with plant proteins often results in reduced loaf volume and poorer texture unless hydrocolloids or other structuring agents are used [29]. Casein micelles stabilize emulsions and form gels in dairy analogues; no single plant protein offers equivalent multifunctionality [30]. Moreover, the functionality of ingredients is context-dependent—the same protein isolate may behave differently in beverages or baked goods, depending on factors such as pH, ionic strength or temperature. Therefore, substitution strategies based solely on composition or single-function analogues often fail to replicate the desired performance.

#### 3.2.2. Predictive and AI-Based Modeling Approaches

Traditional empirical trials tend to overlook the relationships between ingredient concentration, matrix structure and product behavior. To address this issue, rheological modelling, multiscale simulations and machine learning-based prediction are increasingly being used to predict the outcomes of ingredient substitutions. These methods enable formulation designers to simulate functional performance prior to experimentation, thereby reducing trial time and cost [31].

#### 3.2.3. Functional-Nutritional Interactions and Constraints

Inevitably, functional substitutions influence both sensory perception and nutritional value. For example, replacing animal fats with plant-derived oils can modify texture and flavour profiles, while potentially diminishing the bioavailability of fat-soluble vitamins and polyphenols due to differences in emulsion structure and lipid digestion kinetics. In addition, regulatory frameworks—such as restrictions on stabilizers and specific labelling requirements for processing aids—must be observed, often limiting the practical use of otherwise promising ingredient alternatives.

#### 3.2.4. Toward Multifunctional and Clean-Label Solutions

There has recently been a focus on multifunctional natural ingredients (e.g., pea protein, konjac glucomannan, and citrus fibres) that deliver acceptable performance across multiple roles while maintaining clean-label compliance [32]. These ingredients exemplify the convergence of functionality, naturalness and regulatory acceptability, paving the way for sustainable, AI-assisted substitution design.

### 3.3. Nutritional Equivalence and Bioavailability

Nutritional equivalence is a critical factor in ingredient substitution, particularly as consumers and regulators now expect alternatives to preserve—or even enhance—the healthfulness of original products. While macronutrient balancing (e.g., replacing animal fats with plant oils or dairy proteins with legumes) is conceptually straightforward, true nutritional equivalence extends beyond quantity. It also involves bioavailability, nutrient–nutrient interactions, digestibility, and broader health outcomes [33]. One of the most significant challenges, particularly in plant-based reforms, is the variability in protein quality. Plant-based proteins are generally less digestible and offer less favorable amino acid profiles than their animal-derived counterparts, especially in essential amino acids such as lysine and methionine [34]. Although blending strategies can improve amino acid completeness, antinutritional factors—including phytates and tannins—can inhibit protein utilization unless they are adequately removed or offset through processing [35]. Micronutrient substitution presents additional complexity. Animal-derived foods such as red meat and dairy are rich in highly bioavailable forms of iron, calcium, and vitamin B12. In contrast, many plant-based alternatives lack these nutrients unless they are fortified [36]. Moreover, the form of the nutrient significantly influences absorption. For instance, heme iron from animal sources is absorbed more efficiently than non-heme iron from plants. Phytate-rich plant foods can further inhibit mineral uptake [37]. The nutritional functionality of a food also encompasses factors such as fiber content, glycemic index, lipid profile, and the presence of bioactive compounds like polyphenols, peptides, and prebiotics. Without careful consideration of these parameters, substitutions may inadvertently reduce a product’s functional health benefits [38]. AI-based tools have been proposed to support nutrient profiling and formulation optimization. These models integrate ingredient composition data with nutritional databases and dietary guidelines [39]. However, most current implementations still focus mainly on nutrient adequacy, rather than bioefficacy or postprandial metabolic responses. Few models incorporate data on digestive transformations or matrix effects, both of which play a critical role in nutrient release and uptake. Ultimately, nutritional substitution must go beyond label-level matching and adopt a holistic functional nutrition framework. This approach should integrate systems biology, digestive modeling, and, where possible, longitudinal health outcomes to support evidence-based substitution strategies.

### 3.4. Cultural, Religious, and Regulatory Constraints

Ingredient substitution decisions are influenced not only by sensory, functional, or nutritional factors, but also by cultural norms, religious dietary rules, regional expectations, and legal regulations. Ignoring these dimensions can result in substitutions that are technically valid but culturally inappropriate or ethically unacceptable in the target market [40]. Religious dietary frameworks—such as halal (Islamic) and kosher (Jewish)—impose strict rules on permitted ingredients, their sources, and how they are processed. For instance, enzymatic preparations derived from porcine sources or emulsifiers produced via non-certified microbial fermentation may violate compliance, even if they are chemically indistinguishable from acceptable alternatives. Therefore, substitution strategies must account for ingredient traceability and certification compatibility, particularly in global supply chains. Culturally, flavor perception and ingredient familiarity vary widely across regions. Replacing staples such as rice in Asia or olive oil in the Mediterranean with “functionally equivalent” alternatives may result in sensory mismatch or culinary incongruity [41]. This is especially problematic in traditional or ethnic foods, where authenticity is valued as much as nutrition or cost. Regulatory frameworks also impose significant constraints on substitution. These include rules governing ingredient labeling, allergen disclosure, additive thresholds, and approval processes for novel foods. For example, substituting dairy with soy- or nut-based alternatives may trigger allergen labeling requirements [42]. Similarly, emerging protein sources—such as insect flours or mycoproteins—may be unapproved or inconsistently regulated across regions [43]. Some processing aids or enzyme systems may be authorized in one jurisdiction but prohibited in another, complicating efforts to create harmonized global formulations. For instance, the European Food Safety Authority (EFSA) has approved frozen and dried yellow mealworm (Tenebrio molitor larva) as a novel food [44], while in the United States, the FDA regulates edible insects without a standardized premarket framework [45], leading to inconsistent product eligibility and labeling. An often-overlooked dimension is consumer trust and transparency. As AI-generated substitutions and digital formulation tools gain traction, regulators are placing increased emphasis on explainability, documentation, and auditable ingredient provenance [46]. These requirements intersect with broader ethical and legal expectations—including sustainability claims, GMO disclosure, and fair-trade certification. As a result, substitution decisions must be made within a transparent, auditable, and justifiable framework. Integrating cultural, religious, and regulatory constraints into AI-enabled substitution remains a major challenge. Future models must evolve beyond compositional similarity and integrate ontology-aware filtering, traceability logic, and regulatory rule embedding. This will help ensure that substitutions are not only scientifically sound, but also socially, legally, and ethically appropriate.

## 4. AI Methodologies for Ingredient Substitution

Although the use of artificial intelligence (AI) in ingredient substitution is still emerging, recent progress in machine learning (ML), deep learning, graph theory, and natural language processing (NLP) has created new opportunities for solving complex formulation problems. These challenges are typically non-linear, high-dimensional, and involve interdependent variables that exceed the capacity of conventional trial-and-error approaches. AI offers scalable, data-driven alternatives by enabling predictive modeling, automated optimization, and knowledge extraction from large, heterogeneous datasets. Such datasets may contain chemical composition profiles, sensory evaluations, functional metrics, nutritional information, and cultural or consumer preference data. By integrating these diverse inputs, AI systems can identify latent ingredient relationships and generate context-aware substitution recommendations.

### 4.1. Machine Learning for Predictive Modeling

Supervised machine learning models—such as decision trees, support vector machines, and ensemble methods—have been applied to predict functional and nutritional outcomes in ingredient substitution [1,47]. These models are trained on datasets linking ingredient composition, processing conditions, and end-product performance metrics. Regression approaches can estimate properties such as emulsification capacity or protein digestibility from molecular or proximate features [48], while classification models can identify viable substitutes within a given product category [49]. Model performance depends strongly on the quality and completeness of training data, which is often fragmented across proprietary databases, literature sources, and sensory panels. To address data scarcity, strategies such as transfer learning and few-shot learning are increasingly adopted [50]. These models now underpin personalized meal-planning tools and digital formulation assistants that optimize attributes such as texture or allergenicity in near-real-time.

### 4.2. Graph-Based Ingredient Networks

Graph theory provides a structured way to model relationships among ingredients, especially for flavor compatibility, co-occurrence patterns, and functional similarity [51]. In these networks, ingredients act as nodes and their interactions as edges, weighted by chemical similarity, sensory attributes, or recipe co-use frequency. Such graphs allow substitution pathways to be identified by mapping ingredients with comparable properties. Recent work has expanded this approach to heterogeneous graphs that integrate flavor, nutritional, and processing metadata, supporting multi-criteria substitution decisions [1]. Graph embeddings further reduce high-dimensional ingredient relationships into lower-dimensional spaces, enabling unsupervised clustering and the discovery of novel substitutes beyond conventional ingredient groups.

### 4.3. Natural Language Processing (NLP) and Knowledge Mining

With the increasing availability of food-related textual data—such as recipes, consumer reviews, ingredient labels, and scientific papers—natural language processing (NLP) is now widely used to extract semantic relationships among ingredients, functions, and sensory descriptors [52]. Pre-trained language models like BERT and GPT can analyze large datasets to detect patterns in substitution, functional roles, and cultural associations that would be difficult to identify manually. NLP-driven knowledge graphs have been applied to recipe recommendation and question-answering systems, especially when combined with structured resources such as nutrient or sensory databases [53]. The outputs of these models can also feed into predictive pipelines, adding contextual and cultural intelligence to formulation decisions.

### 4.4. Multi-Objective Optimization and Recommender Systems

Ingredient substitution often requires balancing multiple objectives—such as sensory quality, nutritional adequacy, regulatory compliance, and cost. Multi-objective optimization (MOO) techniques, including Pareto optimization, Bayesian methods, and reinforcement learning, have been used to systematically negotiate these trade-offs [54,55]. Recommender systems extend these models into user-facing applications by integrating dietary, sensory, economic, and cultural constraints to generate tailored substitution options. Recent studies show that such systems can identify acceptable ingredient alternatives and that user participation improves adoption rates [56,57]. Platforms like MyFood combine semantic frameworks with neural networks to deliver personalized ingredient and menu suggestions [58]. Current research spans dataset construction, modeling strategies, contextual reasoning, and safety validation, with future work expected to incorporate neuro-symbolic AI and knowledge graphs to improve explainability [1]. Overall, these methods highlight the potential of AI to generate personalized, constraint-aware substitution strategies, provided that sufficient data diversity and integration into food workflows are achieved. Table 1 summarizes the main AI methodologies across sensory, functional, nutritional, and constraint-based dimensions, positioning the proposed framework within the existing research landscape.

## 5. A Modular Framework for AI-Enabled Ingredient Substitution

**Figure 2:**
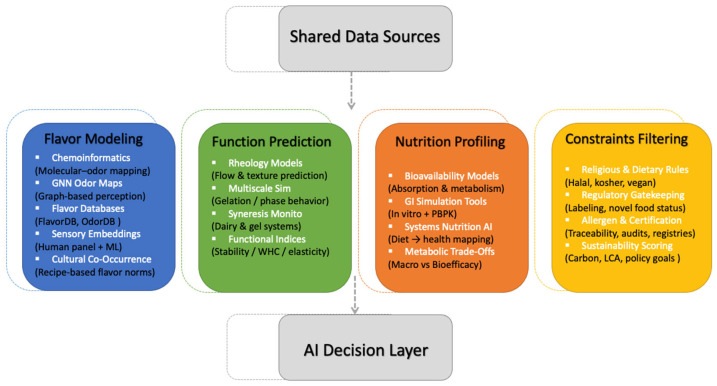
Modular components of the AI-enabled ingredient substitution framework.

Ingredient substitution in modern food design constitutes a multidimensional optimization challenge that extends beyond simple considerations of chemical similarity or ingredient availability. Unlike earlier approaches that treat substitution as a matter of functional matching or flavor mimicry, the framework proposed here integrates sensory science, computational modeling, and regulatory logic into a unified, systems-based decision-making structure. The literature reviewed in this paper lays both the empirical and conceptual groundwork for the modular AI framework proposed here. Research on volatile compound embeddings and flavor-prediction models informs the sensory module, while machine learning studies on rheology, gelation, and structure–function interactions support the functional module. The nutritional module is guided by datasets on nutrient composition, bioavailability, and digestibility, and the constraint module draws on ontology-driven labeling systems, allergen databases, and regulatory filtering tools. Thus, the framework is not introduced as a theoretical abstraction, but as a synthesis of recurring methodological patterns observed across the current body of literature. To address this complexity, we introduce a modular framework that connects AI-based modeling with food system constraints across four interrelated domains: (1) Flavor modeling; (2) Functional property prediction; (3) Nutritional profiling, and (4) Constraint-based filtering. Each module operates semi-independently but contributes to a composite evaluation of substitution suitability, enabling flexible adaptation to specific formulation goals, product categories, or regional market constraints. To enhance conceptual clarity, Figure 2 illustrates the proposed framework, highlighting its four core computational modules and their shared data foundation.

### 5.1. Flavor Modeling

Flavor compatibility is central to consumer acceptance and cannot be determined by molecular similarity alone. This module leverages chemoinformatics, odor network analysis, and machine-learned sensory embeddings to evaluate whether a proposed substitute aligns with target sensory expectations. Databases of volatile compounds (e.g., FlavorDB, OdorDB) and human sensory panel data are increasingly used to train models that predict perceptual outcomes based on molecular input. In addition, graph-based models are applied to analyze ingredient co-occurrence in recipes, capturing both cultural expectations and regional flavor norms. Recent studies have made substantial progress in predicting olfactory perception from molecular structure. Graph neural networks (GNNs) have proven particularly powerful for constructing odor maps and forecasting odor quality [59]. For example, the Principal Odor Map (POM) developed by Lee et al. [60] achieves human-level accuracy in odor description and generalizes well across various prediction tasks. It also outperforms traditional chemoinformatic models by capturing perceptually meaningful relationships among compounds. Earlier work by Keller et al. [61] similarly demonstrated that machine learning algorithms could accurately predict odor intensity, pleasantness, and semantic descriptors from molecular features. These advancements in quantitative structure–odor relationship (QSOR) modeling have far-reaching implications for nutrition science, sensory evaluation, and personalized food design. In the context of ingredient substitution, such models enable more precise selection of aroma analogues that conform not only to chemical similarity but also to human perception and cultural flavor norms. These tools offer a path toward more robust, culturally relevant, and explainable substitution strategies.

### 5.2. Functional Property Prediction

The functionality module simulates the impact of ingredient substitution on key processing attributes—such as emulsification, water binding, and gelation—under specific matrix and process conditions. It integrates rheological modeling, physics-informed neural networks, and multiscale simulations to assess how substitutions alter system behavior. Predictive tools in this domain estimate phase behavior, structural integrity, and the risk of syneresis in various product categories. These models draw from data sources including high-throughput rheology, microstructure imaging, and time–temperature profiles. Parallel to these predictions, functional calibration is essential to validate model outputs. Functional indices—such as emulsion stability, viscoelasticity, and water-holding capacity—serve as critical benchmarks for evaluating simulation accuracy and formulation performance. Recent research has focused on applying advanced imaging and analysis techniques to predict and monitor syneresis, particularly in dairy products like yogurt and cheese [62,63]. These predictions are often calibrated against experimental measures such as the emulsion stability index or dough elasticity, providing feedback loops for improved model reliability and substitution efficacy.

### 5.3. Nutritional Profiling and Digestive Simulation

Nutritional evaluation should extend beyond macronutrient replacement. This module applies systems nutrition models that account for bioavailability, nutrient–nutrient interactions, and the influence of the food matrix on digestion. By integrating dietary reference databases (e.g., USDA, EFSA) with simulation tools—such as in vitro digestion systems and physiologically based absorption models—the framework enables nutrient profiling that aligns with real metabolic outcomes. AI techniques are increasingly used to model the complex, nonlinear relationships between dietary intake and health outcomes. These models support both personalized and population-scale nutritional optimization [64]. In food science, AI has been applied to tasks such as identifying immunity-supporting foods, assessing dietary intake, profiling gut microbiomes, and predicting the toxicity of food ingredients [11]. AI algorithms are also useful in metabolomics, where they help interpret nonlinear interactions between nutrition-related data and physiological outcomes. Technologies like image recognition can enhance dietary assessments by reducing reliance on self-reported intake. In parallel, AI is being used to mine social media data to better understand dietary behaviors and consumer perceptions [65]. While these approaches show great promise, further research is needed to determine where AI delivers added value over traditional nutritional assessment methods. Such models are particularly valuable in ingredient substitution scenarios, where trade-offs between macronutrient composition and bioefficacy must be carefully optimized.

### 5.4. Constraint Filtering: Cultural, Regulatory, and Sustainability Dimensions

The final filtering module evaluates ingredient substitutions based on non-nutritional constraints, including dietary laws (e.g., Halal, Kosher, vegan), regional regulatory frameworks, labeling requirements, and life cycle sustainability impacts. Ontology-based filtering tools and rule-based AI engines can enforce compliance with certification criteria and regional ingredient restrictions. Ontology-aware systems encode rules from religious dietary standards, allergen registries, and certification schemes, allowing AI models to flag substitutions that may be functionally valid but contextually inappropriate. For example, replacing gelatin with agar must satisfy both functional performance and religious compliance criteria [66]. Incorporating AI models with carbon footprint databases allows systems to optimize for both technical performance and environmental sustainability [67]. These tools facilitate informed trade-offs that align with consumer values and policy goals. Each module in the framework produces probabilistic outputs, which are then synthesized through multi-objective optimization techniques—such as Bayesian inference, fuzzy logic, and reinforcement learning—to resolve conflicting criteria (e.g., an ingredient that is nutritionally optimal but lacks flavor compatibility). The full framework supports iterative refinement informed by consumer sensory feedback, in vitro validation, and regulatory review. As a result, it enables a dynamic formulation pipeline that adapts to changing consumer demands, evolving regulations, and sustainability imperatives.

## 6. Emerging Applications and Domain-Specific Use Cases in AI-Driven Ingredient Substitution

Ingredient substitution represents a central challenge in modern food design. Rising demands for sustainability, dietary personalization, and compliance with regulatory and cultural standards are intensifying the need for intelligent, adaptive substitution strategies. Achieving substitutions that preserve sensory fidelity, functional performance, nutritional adequacy, and socio-ethical acceptability requires addressing a tightly interdependent set of objectives. Although many AI-based approaches have been introduced to support substitution decisions, most remain fragmented and domain-specific, lacking integration across functional areas. A comprehensive and scalable solution is still lacking. To bridge this gap, we propose a conceptual framework for Intelligent Ingredient Substitution Systems (IISS)—a modular, AI-powered architecture designed to support transparent, multi-objective, and context-sensitive decision-making. Unlike conventional models, IISS integrates diverse data streams and predictive tools into a single unified platform, enabling simultaneous consideration of flavor chemistry, matrix functionality, nutrient bioefficacy, cultural acceptability, and environmental sustainability. Each module within the IISS corresponds to a distinct, yet interconnected domain, allowing for scalable, system-wide optimization across the entire substitution decision space.

### 6.1. How Can We Ensure Ingredient Substitutions Maintain Sensory Quality, Especially in Aroma, Flavor, and Texture?

#### 6.1.1. The Challenge

Among the many complexities of ingredient substitution, preserving sensory attributes—particularly aroma, flavor, and texture—remains one of the most difficult tasks to address systematically. Flavor and texture are primary determinants of consumer acceptance, yet substitutions often disrupt volatile profiles and structural mouthfeel, leading to noticeable sensory mismatches. These disruptions arise from the nonlinear, matrix-dependent nature of perception, where even subtle compositional changes can have disproportionate sensory effects.

#### 6.1.2. AI Solution

To address this, AI models are increasingly used to simulate and optimize sensory outcomes. Graph-based flavor networks and chemoinformatic models are being coupled with neural sensory embeddings to predict aroma compatibility and volatility behavior. In particular, graph neural networks (GNNs) have shown promise in modeling human olfactory responses from molecular structures. For example, in a landmark study, Lee B. et al. [59] developed a Principal Odor Map (POM) using GNNs trained on thousands of odorant molecules. On a validation set of 400 unseen compounds, the POM’s predicted odor profiles aligned with human panel averages more closely than the median panelist—indicating human-level predictive accuracy. This represents a significant advance in in silico aroma prediction, enabling developers to screen ingredient candidates for aroma fidelity prior to experimental trials. More recently, Tom et al. [68] extended this approach to odorant mixtures, using attention-based POM variants to model the synergistic and antagonistic interactions that occur in real-world food systems. For texture prediction, machine learning models trained on rheological datasets simulate the viscoelastic behavior of food matrices under various processing conditions. These models can predict how substitutions will affect structural attributes such as elasticity, cohesiveness, and mouthfeel. For instance, Dahl et al. [69] showed that Random Forest algorithms could accurately estimate both linear and nonlinear rheological properties—such as storage modulus (G′), loss modulus (G″), and yield stress—in biopolymer matrices including plant protein–polysaccharide blends. These predictions offer a scalable, data-driven alternative to experimental prototyping, particularly for products requiring high structural fidelity (e.g., dairy, baked goods, or gel-based systems).

#### 6.1.3. Real-World Example

The AI system used by NotCo, known as Giuseppe, integrates molecular modeling, sensory data mining, and cultural co-occurrence analysis to develop plant-based analogues for products such as milk, mayonnaise, and ice cream. By combining deep learning with food pairing algorithms, Giuseppe identifies ingredient combinations that successfully replicate both the flavor and texture profiles of conventional formulations.

#### 6.1.4. Remaining Limitations

Despite rapid progress, current models are limited by the lack of open, sensory-labeled datasets, particularly from non-Western and traditional food systems. Additionally, many predictive models are trained on isolated compounds or simplified matrices, which restricts their generalizability to real-world, multi-component food systems. Future work should focus on integrating sensory AI with dynamic matrix modeling and cross-modal interaction data to enable more holistic and culturally adaptive substitution strategies.

### 6.2. How Can We Substitute Functional Ingredients Without Compromising Processability and Product Stability?

#### 6.2.1. The Challenge

Functional substitution is particularly challenging when the target ingredient plays a technological role, such as emulsification, foaming, gelation or viscosity control. Replacements must match the chemical composition, as well as the rheological and structural behavior that determine product stability. In practice, achieving such equivalence through trial and error alone is rarely possible, since changes to the formulation may lead to phase separation, syneresis or texture loss, thereby increasing the time and cost of development.

#### 6.2.2. AI Solution

Artificial intelligence enables in silico prediction of functional behavior before physical prototyping. Machine learning-based rheological models can estimate key parameters such as viscosity, elasticity, and yield stress. For example, Dahl et al. [69] demonstrated that Random Forest algorithms trained on plant protein–polysaccharide blends successfully predicted linear viscoelastic behavior, enabling rapid screening of candidate substitutes for texture fidelity. Similarly, Lee et al. [70] developed ML models to estimate viscosity profiles in hydrocolloid systems, achieving strong correlation with empirical data across concentration, temperature, and shear-rate ranges. To increase industrial applicability, researchers now combine ML tools with physics-informed neural networks (PINNs) and multiscale simulations, bridging the gap between compositional inputs and dynamic functional performance during real-world processing.

#### 6.2.3. Real-World Example

Startups such as Climax Foods and Perfect Day have developed proprietary AI platforms that simulate the behavior of plant-based proteins and fats under various industrial conditions—including heating, cooling, extrusion, and fermentation. These platforms integrate historical performance data, sensory results, and textural analytics to guide formulation strategies. As a result, they can replicate key features such as melting, creaminess, and aeration, particularly in ice cream and cheese analogues. Notably, these AI systems often incorporate upcycled or underutilized plant ingredients, aligning functional substitution with goals related to sustainability and the circular economy.

#### 6.2.4. Remaining Limitations

Despite promising advances, current models still struggle to capture nonlinear mechanical behaviors such as fracture or yield under high strain. Their generalizability across product categories—for example, from beverages to baked goods—remains limited. Moreover, most systems fail to integrate real-time process parameters like shear rate, pH, or thermal gradients, which are crucial for accurate predictions during industrial production. Regulatory compliance adds further complexity, as many models do not consider additive thresholds or clean-label constraints. Finally, a lack of model interpretability poses a barrier—especially in contexts where formulation decisions must be auditable and transparent for regulators or consumers.

### 6.3. How Can We Achieve Nutritional Equivalence in Ingredient Substitution Without Compromising Bioavailability and Health Outcomes?

#### 6.3.1. The Challenge

Although ingredient substitutions are often motivated by goals such as sustainability, allergen reduction, or ethical dietary preferences, they can inadvertently compromise nutritional quality. While macronutrient replacement (e.g., protein-for-protein) is conceptually straightforward, achieving true nutritional equivalence requires addressing far more complex variables—including bioavailability, digestibility, micronutrient density, antinutritional factors, and postprandial metabolic responses. For instance, replacing animal-based proteins or fortified ingredients with plant-based alternatives may reduce levels of critical nutrients such as iron, calcium, and vitamin B12, particularly if the substitutes are unfortified or have low absorption efficiency. Traditional formulation strategies often fail to account for these nuanced interactions, and human clinical trials are generally impractical in early-stage product development.

#### 6.3.2. AI Solution

Artificial intelligence is increasingly being leveraged to address the multi-dimensional challenge of nutritional equivalence. Large language models (LLMs) have shown promise in enhancing phytochemical diversity through ingredient substitutions, thereby supporting healthier dietary patterns [71]. Additionally, knowledge graphs combined with word embeddings can generate substitutability heuristics—allowing for the identification of nutritionally suitable replacements that also respect dietary restrictions, allergen sensitivities, and cultural norms [7]. In the context of precision nutrition, AI-based models have been developed to estimate nutrient intake from dish names and portion sizes, showing high concordance with national dietary survey data [60]. These advances in computational gastronomy aim to unify flavor compatibility, functional roles, and nutritional suitability within a single predictive framework [1]. However, caution is warranted, as many health benefit claims from these models are still based on preclinical data and require further validation for physiological relevance [71].

#### 6.3.3. Real-World Example

Digestibility prediction tools are increasingly being incorporated into ingredient substitution workflows, allowing developers to assess protein quality and nutrient uptake potential. For example, ML models trained on amino acid profiles and protein structure data can simulate the bioefficacy of plant-based protein blends, offering a cost-effective and scalable alternative to in vivo testing—particularly in gluten-free or vegan product development. Moreover, image-based dietary intake assessment platforms are being piloted to deliver real-time nutritional feedback, facilitating faster iteration during early-stage product design without relying on resource-intensive clinical trials.

#### 6.3.4. Remaining Limitations

Despite recent progress, most current models are heavily focused on protein digestion, while analogous tools for micronutrient bioavailability (e.g., iron, zinc, calcium) remain underdeveloped. Digestibility models also rely on well-characterized protein datasets, limiting their applicability to novel or composite ingredients. Additionally, existing frameworks often overlook matrix-specific interactions, antinutritional compounds (e.g., phytates, oxalates), and long-term metabolic outcomes, such as glycemic response or microbiome modulation. Future research must bridge these gaps by integrating mechanistic predictions with epidemiological data and systems biology models—moving beyond basic nutrient matching toward predictive health impact modeling at both the individual and population levels.

### 6.4. How Can Ingredient Substitutions Respect Cultural Norms, Religious Constrains, Regulatory Frameworks, and Sustainability Goals?

#### 6.4.1. The Challenge

Ingredient substitutions that are technically feasible may still fail in practice due to cultural mismatches, religious dietary laws (e.g., Halal, Kosher), legally restricted additives, or undesirable environmental impacts. Navigating diverse certification standards, labeling regulations, and life cycle transparency requirements—especially across global supply chains—adds substantial complexity to formulation strategies.

#### 6.4.2. AI Solution

Ontology-aware filtering systems and rule-based AI engines have emerged as powerful tools to address these multi-dimensional constraints. These systems can parse structured datasets containing ingredient taxonomies, religious dietary rules, allergen registries, and regional regulatory codes to automatically flag non-compliant substitutions. When integrated with life cycle assessment (LCA) frameworks and multi-objective optimization (MOO) algorithms, these models help identify ingredient options that align with regulatory compliance, ethical standards, and environmental objectives. For example, Sunmola et al. [72] developed an AI-powered rule engine that incorporates a global Halal food ontology and supply chain metadata to automatically identify non-compliant ingredient substitutions. Asadollahi et al. [73] proposed a combined MOO-LCA framework to guide sustainable product design, balancing environmental impacts, cost, and material functionality. Similarly, Miranda-Ackerman and Azzaro-Pantel [74] applied carbon labeling and organic content thresholds within a MOO approach to improve environmental performance in green supply chain networks. Rohmer et al. [75] extended these efforts by incorporating sourcing, processing, and logistical factors into substitution models, while ensuring nutritional adequacy—demonstrating the potential of AI in integrating dietary, environmental, and economic dimensions of substitution.

#### 6.4.3. Real-World Example

Commercial platforms such as Plant Jammer, Spoonshot, and HowGood now integrate regulatory filters and environmental metrics into their ingredient recommendation engines. For instance, Plant Jammer allows food manufacturers to prioritize Halal-certified texturizers, exclude allergens, and minimize carbon footprint—all within a guided formulation environment.

#### 6.4.4. Remaining Limitations

Despite growing interest, ontology-based systems depend on well-structured, standardized regulatory datasets, which often differ in format, semantic granularity, and regional specificity. LCA databases are frequently region-bound, limiting the comparability of environmental performance across global contexts. Moreover, underrepresented and dynamic sustainability indicators—such as biodiversity loss, soil degradation, or water scarcity—are rarely incorporated due to data scarcity. Advancing these systems will require more granular, harmonized ontologies, expanded environmental datasets, and greater model transparency, enabling product developers and regulators to understand not only what substitutions are allowed, but why they are selected.

## 7. Key Insights Emerging from the Review

The reviewed literature points to four main developments that are redefining the role of AI in ingredient substitution:

**(1) Substitution is shifting from single-attribute replacement to multidimensional formulation.** Early work largely targeted one variable—most often flavor. Recent studies, however, increasingly treat substitution as an optimization problem that balances sensory, functional, nutritional, and cultural constraints within the same design space.

**(2) Predictive modeling is extending deeper into physicochemical functionality.** Machine learning approaches—including Random Forests, Support Vector Machines, and feedforward neural networks—are now being used to forecast key techno-functional properties such as viscosity, gel strength, emulsification capacity, and rheological behavior of alternative matrices.

**(3) AI is enabling substitutions that are both culturally sensitive and regulation-aware.** Natural-language-based culinary embeddings, graph models, and rule-based systems are being used to recommend alternatives that satisfy dietary laws, allergen restrictions, and culturally specific ingredient norms.

**(4) The primary obstacle to broader model generalization is the absence of standardized datasets.** Across all domains—sensory, functional, nutritional, and contextual—the literature consistently identifies the lack of shared multimodal datasets linking compositional data, processing variables, and sensory outcomes as a central bottleneck.

## 8. Discussion, Future Perspectives and Research Directions

Before exploring avenues for future work, it is essential to critically assess the current limitations of AI-enabled ingredient substitution and to clarify how the proposed framework advances the field. Although recent studies have made notable progress in areas such as flavor prediction, functional modeling, and nutritional optimization, most AI-based substitution systems are still constrained by fragmented datasets, limited model interpretability, and insufficient validation under real-world processing conditions. Unlike previous reviews—many of which focus narrowly on flavor pairing, plant protein texturization, or recipe-level substitutions—this paper frames ingredient substitution as a multi-objective design challenge that spans sensory, functional, nutritional, and socio-regulatory considerations. Nevertheless, despite its integrative structure, the modular framework proposed here operates within several constraints: available datasets remain domain-specific rather than multimodal; most models fail to capture real-time dynamics of manufacturing or digestion; explainability and regulatory alignment are insufficiently addressed; industrial uptake is limited, with many studies still at proof-of-concept stages. Addressing these gaps is crucial for moving beyond theoretical architectures toward fully operational and scalable substitution systems.

As artificial intelligence continues to advance across scientific and industrial domains, its role in food ingredient substitution is poised to grow significantly. However, the field remains nascent, and several key challenges must be addressed to realize AI’s full potential in enabling intelligent, reliable, and ethically grounded substitution decisions.

One major limitation is the lack of comprehensive, multimodal datasets that integrate sensory attributes, functional properties, nutritional composition, allergenicity, cultural acceptance, and sustainability indicators. Most existing models are trained on narrow, domain-specific datasets—such as rheological measurements or aroma compound libraries—without capturing the full complexity of real-world substitution scenarios. Developing standardized, open-access datasets that link ingredient composition with processing parameters and human sensory or nutritional outcomes will be critical. Cross-sectoral data-sharing platforms, potentially governed by consortia of academic, industrial, and regulatory stakeholders, could play a pivotal role in this effort.

Another critical research frontier involves the development of explainable AI (XAI) systems tailored to ingredient-level decision-making. While deep neural networks offer high predictive performance, they often function as black boxes, limiting transparency in highly regulated domains such as food and nutrition. Future work should emphasize interpretable architectures, including attention-based models and graph-based explanation mechanisms, to clarify why specific substitutions are recommended and under what constraints. Enhanced transparency will help build consumer trust, enable third-party audits, and facilitate regulatory compliance.

A further opportunity lies in the real-time deployment of AI within manufacturing environments. While most current systems operate offline and support early-stage formulation, next-generation models should interface dynamically with process sensors, quality control systems, and real-time supply chain data to enable continuous optimization during production. This will require integration with Industry 4.0 technologies, including the Internet of Things (IoT), edge computing, and cyber-physical systems.

Equally important is the need to incorporate personalization and cultural contextualization into substitution strategies. As precision nutrition advances, AI systems must increasingly account for individual dietary restrictions, metabolic profiles, and regional food norms. Models that incorporate clinical nutrition data, wearable health metrics, and local culinary preferences can support inclusive, customized food design. However, such personalization raises concerns around data privacy, algorithmic bias, and governance—issues that necessitate proactive ethical oversight.

Finally, environmental sustainability must be more comprehensively embedded into AI-driven substitution frameworks. While some models incorporate life cycle assessment (LCA) data, many focus narrowly on carbon footprint or energy use. There is an urgent need to expand substitution criteria to include multi-dimensional sustainability indicators, such as water use, biodiversity loss, and land-use change—all of which are central to the transformation of global food systems. Achieving globally applicable models will also require harmonizing sustainability standards across regions and embedding these metrics into the multi-objective optimization logic of AI systems.

In summary, the future of AI-enabled ingredient substitution will depend not only on technical advancements, but also on the creation of a transparent, ethical, and interdisciplinary ecosystem. Realizing this vision will require sustained collaboration among food scientists, AI engineers, nutritionists, sociologists, and policy makers. By uniting these perspectives, the next generation of intelligent systems can move beyond functional reformulation toward a holistic rethinking of ingredient design—supporting a food future that is sustainable, culturally resonant, and nutritionally equitable.

## 9. Conclusions

This review has synthesized current advances in AI-enabled ingredient substitution and evaluated their implications across sensory, functional, nutritional, and regulatory dimensions. Ingredient substitution is no longer a peripheral concern in food science—it has become a central, multidimensional challenge that intersects with sustainability, health, sensory science, technological functionality, cultural norms, and regulatory compliance. Traditional substitution strategies—often reliant on heuristics, empirical testing, and single-objective optimization—are increasingly inadequate in addressing the growing complexity of modern food design. In this evolving landscape, artificial intelligence (AI) emerges as a transformative enabler, reframing ingredient substitution as a systems-level optimization problem, guided by data-rich, context-aware, and multi-objective reasoning. This review has systematically examined the application of AI in ingredient substitution across five interrelated domains: sensory fidelity, functional performance, nutritional equivalence, socio-regulatory compliance, and environmental sustainability. We demonstrated that machine learning, graph-based modeling, natural language processing, and ontology-aware systems each offer unique contributions to the development of intelligent and scalable substitution frameworks. Real-world examples—from sensory emulators and digestibility predictors to compliance engines—underscore the practical utility of these tools, while also highlighting persistent challenges in model interpretability and cross-domain integration. To transition from experimental prototypes to widespread industrial adoption, future AI-enabled substitution systems must prioritize explainability, cultural inclusiveness, regulatory robustness, and ecological accountability. The proposed modular framework for Intelligent Ingredient Substitution Systems (IISSs) provides a conceptual foundation for integrating these priorities into a cohesive, actionable platform. Achieving this vision will require sustained interdisciplinary collaboration, transparent data governance, and a commitment to aligning algorithmic intelligence with human values. Ultimately, the convergence of food science and AI holds profound potential—not merely to reformulate recipes, but to reimagine how we design, evaluate, and justify ingredients in a rapidly changing world. In doing so, we may advance toward a food system that is not only technologically advanced, but also sustainable, equitable, and resilient in the face of global challenges.

## Figures and Tables

**Table 1:** AI methodologies for ingredient substitution: algorithms, applications, and performance highlights.

Algorithm Type	Application Area	Main Performance Indicators/Highlights	Representative Studies
Machine Learning (SVM, Decision Trees, Ensembles)	Functional and nutritional prediction; modeling of physicochemical or emulsification behavior	Models nonlinear relationships between compositional or molecular features and functional outcomes; enables predictive formulation and personalized nutrition.	[11,47,48,49]
Graph-based Models (Graph Theory, Graph Neural Networks)	Flavor compatibility/ingredient relationship modeling	Represents ingredients as nodes and interactions as edges; graph embeddings support unsupervised learning and multi-criteria substitution mapping.	[1,51]
Natural Language Processing (NLP) and Knowledge Mining	Text-based recipe, label, and review analysis; context-aware ingredient substitution	Extracts semantic relations among ingredients, sensory descriptors, and cultural associations; supports knowledge-graph-based recommendation systems.	[52,53]
Multi-objective Optimization (Bayesian Optimization, Pareto Front, Reinforcement Learning)	Balancing sensory, nutritional, regulatory, and economic constraints in formulation	Systematically optimizes multiple competing objectives; forms the basis for recommender systems generating acceptable substitutions.	[54,55,56,57]
Constraint-aware and Ontology-based AI Engines (within the IISS Framework)	Cultural, regulatory, and sustainability filtering of substitutions	Applies rule-based and ontology-aware reasoning to ensure dietary, labeling, and environmental compliance within AI substitution pipelines.	[1,58]

## Data Availability

No new data was used for the research. Data sharing is not applicable to this article.

## References

[1] Kim H., Venkataramanan R., Sheth A. (2024). A survey on food ingredient substitutions. arXiv.

[2] Tachie C., Nwachukwu I.D., Aryee A.N. (2023). Trends and innovations in the formulation of plant-based foods. Food Prod. Process. Nutr..

[3] Karabulut G., Goksen G., Khaneghah A.M. (2024). Plant-based protein modification strategies towards challenges. J. Agric. Food Res..

[4] Davies K.P., Gibney E.R., O’Sullivan A.M. (2023). Moving towards more sustainable diets: Is there potential for a personalised approach in practice?. J. Hum. Nutr. Diet..

[5] Groetch M., Skypala I. (2023). When nutrition trends and food allergies collide. Ann. Allergy Asthma Immunol..

[6] Edge M.S. (2020). The balancing act—Nutrition and sustainability: Understanding the complexities, challenges, and opportunities. Nutr. Today.

[7] Shirai S.S., Seneviratne O., Gordon M.E., Chen C.H., McGuinness D.L. (2021). Identifying ingredient substitutions using a knowledge graph of food. Front. Artif. Intell..

[8] Zhang X., Zhou T., Zhang L., Fung K.Y., Ng K.M. (2019). Food product design: A hybrid machine learning and mechanistic modeling approach. Ind. Eng. Chem. Res..

[9] Kumar I., Rawat J., Mohd N., Husain S. (2021). Opportunities of artificial intelligence and machine learning in the food industry. J. Food Qual..

[10] Aghababaei A., Aghababaei F., Pignitter M., Hadidi M. (2025). Artificial intelligence in agro-food systems: From farm to fork. Foods.

[11] Miyazawa T., Hiratsuka Y., Toda M., Hatakeyama N., Ozawa H., Abe C., Miyazawa T. (2022). Artificial intelligence in food science and nutrition: A narrative review. Nutr. Rev..

[12] Kansaksiri P., Panomkhet P., Tantisuwichwong N. (2023). Smart cuisine: Generative recipe & ChatGPT powered nutrition assistance for sustainable cooking. Procedia Comput. Sci..

[13] Cui Z., Qi C., Zhou T., Yu Y., Wang Y., Zhang Z., Liu Y. (2025). Artificial intelligence and food flavor: How AI models are shaping the future and revolutionary technologies for flavor food development. Compr. Rev. Food Sci. Food Saf..

[14] Dhal S.B., Kar D. (2025). Leveraging artificial intelligence and advanced food processing techniques for enhanced food safety, quality, and security: A comprehensive review. Discov. Appl. Sci..

[15] Jakkan S., Patil R.S., Ekad S., Shinde P. (2025). Innovations in recipe generation and ingredient substitution: A survey on RAG and Generative AI approaches. Int. J. Innov. Sci. Res. Technol..

[16] Buyuktepe O., Catal C., Kar G., Bouzembrak Y., Marvin H., Gavai F. (2025). Food fraud detection using explainable artificial intelligence. Expert Syst..

[17] Arrighi L., de Moraes I.A., Zullich M., Simonato M., Barbin D.F., Junior S.B. (2025). Explainable Artificial Intelligence techniques for interpretation of food datasets: A review. arXiv.

[18] Mallela I.R., Aravind S., Salunkhe V., Taharan O., Goel P., Singh S.P. (2020). Explainable AI for compliance and regulatory models. Int. J. Innov. Sci. Res. Technol..

[19] Bura C., Jonnalagadda A.K., Naayini P. (2024). The role of Explainable AI (XAI) in trust and adoption. J. Artif. Intell. Gen. Sci. (JAIGS).

[20] Sarkar M., Rashid M.H.O., Hoque M.R., Mahmud M.R. (2025). Explainable AI in e-commerce: Enhancing trust and transparency in AI-driven decisions. Innov. Eng. J..

[21] Li Y., Zhang Q., Liu X., Bian X., Li J., Meng N., Li J. (2025). Flavor interactions in wine: Current status and future directions from interdisplinary and crossmodal perspectives. Compr. Rev. Food Sci. Food Saf..

[22] McClintock T.S., Wang Q., Sengoku T., Titlow W.B., Breheny P. (2020). Mixture and concentration effects on odorant receptor response patterns in vivo. Chem. Senses.

[23] Frank D.C., Eyres G.T., Piyasiri U., Delahunty C.M. (2012). Effect of food matrix structure and composition on aroma release during oral processing using in vivo monitoring. Flavour Fragr. J..

[24] Martínez-Mayorga K., Peppard T.L., Yongye A.B., Santos R., Giulianotti M., Medina-Franco J.L. (2011). Characterization of a comprehensive flavor database. J. Chemom..

[25] Rivero-Pino F., Millan-Linares M.C., Montserrat-De-La-Paz S. (2023). Strengths and limitations of in silico tools to assess physicochemical properties, bioactivity, and bioavailability of food-derived peptides. Trends Food Sci. Technol..

[26] Boscarino C., Nedović V., Koenderink N.J., Top J.L. Automatic extraction of ingredient’s substitutes. Proceedings of the 2014 ACM International Joint Conference on Pervasive and Ubiquitous Computing: Adjunct Publication.

[27] Sauer C., Haigh A., Rachleff J. (2017). Cooking up Food Embeddings. Understanding Flavors in the Recipe-Ingredient Graph. https://snap.stanford.edu/class/cs224w-2017/projects/cs224w-34-final.pdf.

[28] Phillips L.G. (2013). Structure-Function Properties of Food Proteins.

[29] Gómez M., Sciarini L.S., Arranz E., Fernández-Bañares F., Rosell C.M., Rodrigo L., Peña A.S. (2015). Gluten-Free Bakery Products and Pasta. Advances in the Understanding of Gluten Related Pathology and the Evolution of Gluten-Free Foods.

[30] Tang Q., Roos Y.H., Miao S. (2024). Structure, gelation mechanism of plant proteins versus dairy proteins and evolving modification strategies. Trends Food Sci. Technol..

[31] Chitra Devi V., Devanampriyan R., Kayethri D., Sankari R., Premalatha J., Sathish Raam R., Mothil S. (2025). Optimization and process validation of freeze-structured meat substitute using Machine Learning Models. J. Food Process Eng..

[32] Polachini T.C., Norwood E.A., Le-Bail P., Le-Bail A. (2023). Clean-label techno-functional ingredients for baking products–a review. Crit. Rev. Food Sci. Nutr..

[33] Simpson S.J., Le Couteur D.G., Raubenheimer D. (2015). Putting the balance back in diet. Cell.

[34] Gorissen S.H., Witard O.C. (2018). Characterising the muscle anabolic potential of dairy, meat and plant-based protein sources in older adults. Proc. Nutr. Soc..

[35] Gilani G.S., Xiao C.W., Cockell K.A. (2012). Impact of antinutritional factors in food proteins on the digestibility of protein and the bioavailability of amino acids and on protein quality. Br. J. Nutr..

[36] Yeo M.T.Y., Bi X., Henry C.J. (2023). Are plant-based meat analogues richer in minerals than their meat counterparts?. Food Humanit..

[37] Kapil R. (2017). Bioavailability & absorption of iron and anemia. Indian J. Community Health.

[38] Perez-Gregorio R., Simal-Gandara J. (2017). A critical review of bioactive food components, and of their functional mechanisms, biological effects and health outcomes. Curr. Pharm. Des..

[39] Sharma S.K., Gaur S. (2024). Optimizing nutritional outcomes: The role of AI in personalized diet planning. Int. J. Res. Publ. Semin.

[40] Lindgreen A., Hingley M.K. (2010). The new cultures of food: Marketing opportunities from ethnic religious and cultural diversity. J. Consum. Mark..

[41] Lee C.L., Lee S.H., Seo G.G., Hong J.H. (2020). The effect of plating, ingredients, and cooking processes on the acceptance and authenticity of ethnic rice dishes. Foods.

[42] EFSA Panel on Nutrition, Novel Foods and Food Allergens (NDA) (2021). Turck, D.; Bohn, T.; Castenmiller, J.; De Henauw, S.; Hirsch-Ernst, K.I.; Knutsen, H.K. Safety of frozen and dried formulations from whole yellow mealworm (*Tenebrio molitor* larva) as a novel food pursuant to Regulation (EU) 2015/2283. EFSA J..

[43] Lähteenmäki-Uutela A., Marimuthu S.B., Meijer N. (2021). Regulations on insects as food and feed: A global comparison. J. Insects Food Feed.

[44] EFSA Panel on Dietetic Products, Nutrition and Allergies (NDA) (2014). Scientific opinion on the evaluation of allergenic foods and food ingredients for labelling purposes. EFSA J..

[45] Larouche J., Campbell B., Hénault-Éthier L., Banks I.J., Tomberlin J.K., Preyer C., Vandenberg G.W. (2023). The edible insect sector in Canada and the United States. Anim. Front..

[46] Longpre S., Mahari R., Obeng-Marnu N., Brannon W., South T., Gero K., Pentland S., Kabbara J. Position: Data authenticity, consent, & provenance for AI are all broken: What will it take to fix them? In Proceedings of the 41st International Conference on Machine Learning, Vienna, Austria, 21–27 July 2024; Volume 235.

[47] Tachie C., Tawiah N.A., Aryee A.N. (2023). Using machine learning models to predict the quality of plant-based foods. Curr. Res. Food Sci..

[48] Keivaninahr F., Gadkari P., Benis K.Z., Tulbek M., Ghosh S. (2021). Prediction of emulsification behaviour of pea and faba bean protein concentrates and isolates from structure–functionality analysis. RSC Adv..

[49] Phillips K.A., Wambaugh J.F., Grulke C.M., Dionisio K.L., Isaacs K.K. (2017). High-throughput screening of chemicals as functional substitutes using structure-based classification models. Green Chem..

[50] Parnami A., Lee M. (2022). Learning from few examples: A summary of approaches to few-shot learning. arXiv.

[51] Pyo S.J. (2025). Flavor Diffusion: Predicting food pairings and chemical interactions using diffusion models. arXiv.

[52] Cui H., Yan R., Cao Q., Zhang J. (2025). High-reputation food formulas: A heterogeneous information network representation and semantic analysis approach. Appl. Sci..

[53] Min W., Liu C., Xu L., Jiang S. (2022). Applications of knowledge graphs for food science and industry. Patterns.

[54] Banga J.R., Balsa-Canto E., Alonso A.A. (2008). Quality and safety models and optimization as part of computer-integrated manufacturing. Compr. Rev. Food Sci. Food Saf..

[55] Brochu E., Cora V.M., De Freitas N. (2010). A tutorial on Bayesian optimization of expensive cost functions, with application to active user modeling and hierarchical reinforcement learning. arXiv.

[56] Vandeputte J., Herold P., Kuslii M., Viappiani P., Muller L., Martin C., Darcel N. (2023). Principles and validations of an artificial intelligence-based recommender system suggesting acceptable food changes. J. Nutr..

[57] Chelmis C., Gergin B. Recipe networks and the principles of healthy food on the Web. Proceedings of the International AAAI Conference on Web and Social Media.

[58] Sandri S., Molinari A. Preference learning in food recommendation: The” Myfood” case study. Proceedings of the 2023 3rd International Conference on Electrical, Computer, Communications and Mechatronics Engineering (ICECCME).

[59] Lee B.K., Mayhew E.J., Sanchez-Lengeling B., Wei J.N., Qian W.W., Little K., Wiltschko A.B. (2022). A principal odor map unifies diverse tasks in human olfactory perception. bioRxiv.

[60] Lee H.A., Huang T.T., Yen L.H., Wu P.H., Chen K.W., Kung H.H., Hsu C.Y. (2022). Precision nutrient management using artificial intelligence based on digital data collection framework. Appl. Sci..

[61] Keller A., Gerkin R.C., Guan Y., Dhurandhar A., Turu G., Szalai B., Meyer P. (2016). Reverse-engineering human olfactory perception from chemical features of odor molecules. bioRxiv.

[62] Andersen U., Huang J., Esbensen K.H., Mikkelsen B., Knudsen L.B. (2003). Prediction of syneresis in yoghurt by means of Confocal Laser Scanning Microscopy (CLSM) and image analysis. Fermented Milk: Proceedings of the IDF Seminar on Aroma and Texture of Fermented Milk Held in Kolding, Denmark, June 2002.

[63] Fagan C.C., Du C.J., O’Donnell C.P., Castillo M., Everard C.D., O’Callaghan D.J., Payne F.A. (2008). Application of image texture analysis for online determination of curd moisture and whey solids in a laboratory-scale stirred cheese vat. J. Food Sci..

[64] Knights V., Kolak M., Markovikj G., Gajdoš Kljusurić J. (2023). Modeling and optimization with artificial intelligence in nutrition. Appl. Sci..

[65] Côté M., Lamarche B. (2022). Artificial intelligence in nutrition research: Perspectives on current and future applications. Appl. Physiol. Nutr. Metab..

[66] Mahamud N., Santiworakun N.Y., Chaovasuteeranon S., Boonmalert F. (2023). Halal alternative sources of gelatin: A review. J. Halal Sci. Ind. Bus..

[67] Alle K. (2018). Advancing sustainable AI: Balancing performance and carbon emissions with system of Systems Theory. Comput. Sci. Inf. Technol..

[68] Tom G., Ser C.T., Rajaonson E.M., Lo S., Park H.S., Lee B.K., Sanchez-Lengeling B. (2025). From molecules to mixtures: Learning representations of olfactory mixture similarity using inductive biases. arXiv.

[69] Dahl J.F., Schlangen M., van der Goot A.J., Corredig M. (2025). Predicting rheological parameters of food biopolymer mixtures using machine learning. Food Hydrocoll..

[70] Lee D., Jeong S., Yun S., Lee S. (2024). Artificial intelligence-based prediction of the rheological properties of hydrocolloids for plant-based meat analogues. J. Sci. Food Agric..

[71] Rita L., Southern J., Laponogov I., Higgins K., Veselkov K. (2024). Optimizing ingredient substitution using large language models to enhance phytochemical content in recipes. Mach. Learn. Knowl. Extr..

[72] Sunmola F., Baryansis G., Tan A., Co K., Papadakis E. (2025). Holistic framework for blockchain-based halal compliance in supply chains enabled by artificial intelligence. Systems.

[73] Asadollahi A., Tohidi H., Shoja A. (2022). Integration of a Multi-Objective Optimization Model and Life Cycle Assessment into Sustainable Product Design: A Case Study in Food Packaging. https://ssrn.com/abstract=4041795.

[74] Miranda-Ackerman M.A., Azzaro-Pantel C. (2017). Extending the scope of eco-labelling in the food industry to drive change beyond sustainable agriculture practices. J. Environ. Manag..

[75] Rohmer S.U.K., Gerdessen J.C., Claassen G.D.H. (2019). Sustainable supply chain design in the food system with dietary considerations: A multi-objective analysis. Eur. J. Oper. Res..

